# Supplementary material for: Differential impacts of reduced worktime on work-life balance in Korea
Source: PLoS One. 2023 Nov 16;18(11):e0294247. doi: 10.1371/journal.pone.0294247 (PMC10653494; doi:10.1371/journal.pone.0294247)
Supplement: S2 Table — (DOCX) [file pone.0294247.s002.docx]

S2 Table. Self-selection Check

|  | (1) | (2) | (3) |
| --- | --- | --- | --- |
|  | Job satisfaction | Workhour satisfaction | Leisure satisfaction |
| *Panel A: Workers by gender* |  |  |  |
| Total Workers | 0.060*** (0.010) | 0.043*** (0.010) | 0.013 (0.010) |
| Male Workers | 0.061*** (0.012) | 0.047*** (0.013) | 0.007 (0.012) |
| Female Workers | 0.058*** (0.016) | 0.037** (0.016) | 0.023 (0.015) |
| *Panel B: By gender and education* |  |  |  |
| Male Workers – High School Completion or Less | 0.073*** (0.016) | 0.039**(0.017) | -0.011 (0.016) |
| Male Workers – College or Higher | 0.039**(0.019) | 0.050** (0.020) | 0.023 (0.019) |
| Female Workers – High School Completion or Less | 0.041** (0.019) | 0.034* (0.020) | 0.053*** (0.017) |
| Female Workers – College or Higher | 0.086*** (0.028) | 0.037 (0.029) | -0.022 (0.028) |
| *Panel C: By gender and marital status* |  |  |  |
| Unmarried Male Workers | 0.064** (0.026) | 0.049* (0.027) | 0.027 (0.024) |
| Married Male Workers | 0.056*** (0.015) | 0.052*** (0.015) | 0.002 (0.015) |
| Unmarried Female Workers | 0.076*** (0.028) | 0.048* (0.028) | 0.043 (0.027) |
| Married Female Workers | 0.041** (0.020) | 0.014 (0.021) | 0.016 (0.019) |
| *Panel D: By gender and parental status* |  |  |  |
| Male Workers without Children | 0.055*** (0.019) | 0.032 (0.020) | 0.006 (0.019) |
| Male Workers with Children | 0.066*** (0.017) | 0.064*** (0.018) | 0.006 (0.018) |
| Female Workers without Children | 0.053** (0.023) | 0.035 (0.023) | 0.021 (0.022) |
| Female Workers with Children | 0.051** (0.026) | 0.006 (0.027) | 0.002 (0.024) |
| *Panel E: By gender and precarious employment* |  |  |  |
| Male Workers with Precarious Employment | 0.034 (0.027) | 0.094*** (0.031) | 0.061** (0.027) |
| Male Workers without Precarious Employment | 0.063*** (0.014) | 0.037** (0.015) | -0.004 (0.015) |
| Female Workers with Precarious Employment | 0.024 (0.030) | 0.032 (0.031) | 0.017 (0.025) |
| Female Workers without Precarious Employment | 0.059*** (0.022) | 0.016 (0.022) | 0.028 (0.021) |
| *Panel F: By gender and flexible employment* |  |  |  |
| Male Workers with Flexible Worktime | -0.010 (0.030) | 0.058* (0.031) | 0.050 (0.030) |
| Male Workers without Flexible Worktime | 0.050*** (0.017) | 0.031* (0.018) | -0.014 (0.018) |
| Female Workers with Flexible Worktime | 0.065 (0.044) | 0.080* (0.046) | -0.013 (0.036) |
| Female Workers without Flexible Worktime | 0.051** (0.023) | 0.013 (0.023) | 0.029 (0.021) |
