# Supplementary material for: Differential impacts of reduced worktime on work-life balance in Korea
Source: PLoS One. 2023 Nov 16;18(11):e0294247. doi: 10.1371/journal.pone.0294247 (PMC10653494; doi:10.1371/journal.pone.0294247)
Supplement: S3 Table — (DOCX) [file pone.0294247.s003.docx]

S3 Table. Variable Description

| Variable | Questionnaire | Coding |
| --- | --- | --- |
| Weekly working hours | How many hours do you work in a week excluding meal times? | Numerical  (The number of working hours per week) |
| Working over 40 hours a week | How many hours do you work in a week excluding meal times? | Binary  (The number of working hours per week >40 = 1,  The number of working hours per week =<40 = 0) |
| Workhour satisfaction | How satisfied are you with your working hours in relation to your main job? | Binary  (Satisfied, Very Satisfied = 1, Neutral, Dissatisfied, Very Dissatisfied = 0) |
| Job satisfaction | How satisfied are you overall with your main job? | Binary  (Satisfied, Very Satisfied = 1, Neutral, Dissatisfied, Very Dissatisfied = 0) |
| Leisure satisfaction | How satisfied are you with your leisure activities? | Binary  (Satisfied, Very Satisfied = 1, Neutral, Dissatisfied, Very Dissatisfied = 0) |
| Age | When is your date of birth? | Numerical (20-64 years old) |
| Married | What is your current marital status? | Binary  Married with a spouse = 1  Single, Separated, Divorced, Spouse has passed away = 0 |
| Have children | Do you have any children in your household who are aged 0 or older but are high school students or younger? | Binary  (Yes = 1, No = 0) |
| College level education or above | Until what level of education have you attended? Or are you currently attending?" | Binary  (College level education or above = 1,  Below college level education = 0) |
| Precarious employment | (Main job) Regular/Non-regular employment status | Binary  (Non-regular employment status = 1, Regular employment status = 0) |
| Flexible employment | Is there a set regular working hours for this job currently? | Binary  (No = 1, Yes = 0) |
| GRDP per capita | Gross Regional Domestic Product per capita | Numerical (in 1,000 won) |
