# Supplementary material for: Differential impacts of reduced worktime on work-life balance in Korea
Source: PLoS One. 2023 Nov 16;18(11):e0294247. doi: 10.1371/journal.pone.0294247 (PMC10653494; doi:10.1371/journal.pone.0294247)
Supplement: S4 Table — (DOCX) [file pone.0294247.s004.docx]

S4 Table. Other Satisfaction Measures

|  | (1) | (2) | (3) |
| --- | --- | --- | --- |
|  | Family relationship satisfaction | Housing satisfaction | Social life satisfaction |
| *Panel A: Workers by gender* |  |  |  |
| Total Workers | -0.0004 (0.008) | 0.006 (0.008) | -0.002 (0.009) |
| Male Workers | -0.010 (0.010) | -0.005 (0.011) | -0.004 (0.011) |
| Female Workers | 0.016 (0.013) | 0.022 (0.014) | 0.003 (0.014) |
| *Panel B: By gender and education* |  |  |  |
| Male Workers – High School Completion or Less | 0.005 (0.015) | -0.014 (0.015) | 0.002 (0.016) |
| Male Workers – College or Higher | -0.023 (0.014) | 0.002 (0.016) | -0.008 (0.016) |
| Female Workers – High School Completion or Less | 0.046** (0.018) | 0.015 (0.018) | 0.005 (0.018) |
| Female Workers – College or Higher | -0.022 (0.020) | 0.026 (0.022) | 0.005 (0.023) |
| *Panel C: By gender and marital status* |  |  |  |
| Unmarried Male Workers | -0.034 (0.021) | -0.025 (0.020) | -0.027 (0.022) |
| Married Male Workers | 0.006 (0.012) | 0.002 (0.013) | 0.011 (0.013) |
| Unmarried Female Workers | 0.017 (0.022) | 0.019 (0.022) | 0.0005 (0.023) |
| Married Female Workers | 0.019 (0.018) | 0.015 (0.019) | -0.004 (0.018) |
| *Panel D: By gender and parental status* |  |  |  |
| Male Workers without Children | -0.026 (0.017) | -0.021 (0.016) | -0.006 (0.017) |
| Male Workers with Children | 0.002 (0.014) | 0.008 (0.016) | 0.008 (0.016) |
| Female Workers without Children | 0.013 (0.019) | 0.012 (0.019) | 0.008 (0.020) |
| Female Workers with Children | 0.022 (0.022) | 0.019 (0.022) | 0.001 (0.023) |
| *Panel E: By gender and precarious employment* |  |  |  |
| Male Workers with Precarious Employment | 0.018 (0.029) | 0.050* (0.026) | 0.006 (0.028) |
| Male Workers without Precarious Employment | -0.024** (0.012) | -0.022* (0.013) | -0.007 (0.013) |
| Female Workers with Precarious Employment | 0.010 (0.027) | -0.005 (0.026) | -0.0002 (0.026) |
| Female Workers without Precarious Employment | 0.005 (0.017) | 0.032* (0.018) | -0.004 (0.019) |
| *Panel F: By gender and flexible employment* |  |  |  |
| Male Workers with Flexible Worktime | 0.053* (0.031) | 0.030 (0.029) | 0.046 (0.030) |
| Male Workers without Flexible Worktime | -0.020 (0.014) | -0.010 (0.015) | -0.015 (0.015) |
| Female Workers with Flexible Worktime | 0.070* (0.041) | 0.032 (0.038) | -0.029 (0.041) |
| Female Workers without Flexible Worktime | 0.008 (0.018) | 0.033* (0.019) | 0.005 (0.019) |
