# Supplementary material for: Differential impacts of reduced worktime on work-life balance in Korea
Source: PLoS One. 2023 Nov 16;18(11):e0294247. doi: 10.1371/journal.pone.0294247 (PMC10653494; doi:10.1371/journal.pone.0294247)
Supplement: S5 Table — (DOCX) [file pone.0294247.s005.docx]

S5 Table. Generalized Difference-in-Differences Estimates

|  | (1) | (2) | | (3) |
| --- | --- | --- | --- | --- |
|  | Job satisfaction | Workhour satisfaction | | Leisure satisfaction |
| *Panel A: Workers by gender* | | |  |  |
| Total Workers | 0.034***  (0.012) | 0.032**  (0.015) | | 0.008  (0.012) |
| Male Workers | 0.021  (0.013) | 0.024  (0.016) | | 0.002  (0.016) |
| Female Workers | 0.061***  (0.023) | 0.04*  (0.023) | | 0.014  (0.018) |
| *Panel B: By gender and education* | | |  |  |
| Male Workers – High School Completion or Less | 0.026  (0.019) | 0.003  (0.021) | | -0.016  (0.02) |
| Male Workers – College or Higher | 0.016  (0.024) | 0.05*  (0.026) | | 0.023  (0.025) |
| Female Workers – High School Completion or Less | 0.019  (0.023) | 0.027  (0.026) | | 0.05**  (0.024) |
| Female Workers – College or Higher | 0.119***  (0.034) | 0.064*  (0.037) | | -0.024  (0.036) |
| *Panel C: By gender and marital status* | | |  |  |
| Unmarried Male Workers | 0.036  (0.032) | 0.023  (0.032) | | 0.015  (0.033) |
| Married Male Workers | 0.021  (0.017) | 0.03  (0.018) | | -0.0002  (0.019) |
| Unmarried Female Workers | 0.11***  (0.034) | 0.089**  (0.036) | | 0.01  (0.035) |
| Married Female Workers | 0.039  (0.026) | 0.003  (0.026) | | 0.021  (0.023) |
| *Panel D: By gender and parental status* | | |  |  |
| Male Workers without Children | 0.006  (0.022) | 0.011  (0.026) | | -0.015  (0.026) |
| Male Workers with Children | 0.032  (0.024) | 0.053**  (0.021) | | 0.011  (0.021) |
| Female Workers without Children | 0.087***  (0.031) | 0.071**  (0.028) | | 0.034  (0.026) |
| Female Workers with Children | 0.054  (0.036) | 0.022  (0.036) | | 0.001  (0.031) |
| *Panel E: By gender and precarious employment* | | |  |  |
| Male Workers with Precarious Employment | 0.016  (0.017) | 0.014  (0.019) | | 0.002  (0.021) |
| Male Workers without Precarious Employment | 0.006  (0.039) | 0.035  (0.037) | | 0.002  (0.041) |
| Female Workers with Precarious Employment | 0.04  (0.029) | 0.016  (0.029) | | 0.007  (0.029) |
| Female Workers without Precarious Employment | 0.021  (0.043) | 0.06*  (0.036) | | 0.029  (0.035) |
| *Panel F: By gender and flexible employment* | | |  |  |
| Male Workers with Flexible Worktime | 0.075***  (0.024) | 0.056**  (0.026) | | 0.027  (0.026) |
| Male Workers without Flexible Worktime | -0.045  (0.044) | 0.063  (0.053) | | 0.013  (0.048) |
| Female Workers with Flexible Worktime | 0.059*  (0.03) | 0.038  (0.032) | | 0.007  (0.026) |
| Female Workers without Flexible Worktime | 0.056  (0.057) | 0.103  (0.067) | | 0.052  (0.054) |
