# Supplementary material for: Differential impacts of reduced worktime on work-life balance in Korea
Source: PLoS One. 2023 Nov 16;18(11):e0294247. doi: 10.1371/journal.pone.0294247 (PMC10653494; doi:10.1371/journal.pone.0294247)
Supplement: Attachment — Submitted filename: Rebuttal Letter.docx [file pone.0294247.s006.docx]

*We thank the reviewers for thoughtful comments. We addressed all issues raised by the reviewers by revising the manuscript to incorporate changes described below.*

**Reviewer #1**

- *The authors exclude those working 30 hours or fewer per week. Is it possible to distinguish contracted work hours rather than actual work hours?*

**Response**: The panel data set we used (Korean Labor & Income Panel Study) does not contain information on contracted work hours. We added the following comment in the **Data** section;

“The KLIPS does not provide information on contracted work hours. Therefore, we cannot differentiate work hours and contracted hours in our analysis.”

- *The authors state: “Individual fixed effects (*$\gamma_{i}$*) control for time-invariant and unobservable factors at the individual level.” Does this imply a) that the authors have used a fixed effects regression, analyzing only within individual differences? If yes, specify. If not, clarify.*

**Response**: We specified two-way fixed effects regressions controlling for individual fixed effects and year fixed effects. We clarified this in the **Empirical strategy** section as follows;

“In our two-way fixed effects model, we add individual fixed effects (*γ_i_*) to control for time-invariant and unobservable factors at the individual level.”

- *The questions used for the key variable are missing. I particularly miss the wording of the questions for work hours and satisfaction (translated to English), and if these questions are self-made or developed and tested elsewhere. Furthermore, is satisfaction measured using single items or scales? If scales, please also add alpha values.*

**Response**: We translated the questionnaires of the work hours and satisfaction measures and added them in **Appendix** S3 Table. The following statement is added in the **Variables** section;

“The questionnaires of the work hours and satisfaction measures are translated to English and presented in S3 Table.”

Also, our satisfaction indicators are measured on single items. Specific questionnaires for each indicator are added in the **Variables** section as follows;

“Each satisfaction measure is based on single items, respectively asking “How satisfied are you with your working hours in relation to your main job?”, “How satisfied are you overall with your main job?”, and “How satisfied are you with your leisure activities?”

- *I also miss information on the types of analyses, and how to interpret the coefficients in table 3. Can I assume that model 1 (weekly work hours) is a linear regression, while the rest is logistic regression? And are the coefficients for logistic regression presented OR, average marginal effects or what?*

**Response**: As stated in the **Empirical strategy** section, we specified linear regressions. In order to clarify our methodology, we added a sentence in the **Results** section as follows;

“Note that each column represents the estimates of the two-way fixed effects linear regressions specified in equation (1).”

- *Satisfaction is measured on a 5 point Likert scale, but the authors dichotomize the outcome. Why? Keeping the variation in the outcome is generally preferable, and alternative solutions should be explained.*

**Response**: Following literature, we dichotomized the satisfaction measures, because the extreme measures have smaller cell sizes, so that combining the responses simplifies computational processes. We added the following sentences in the Variables section;

“We dichotomized the satisfaction measures because the extreme measures have smaller cell sizes (all less than 1 percent in our analytic sample). Therefore, combining the responses simplifies computational processes.”

- *Several comparisons are made between different subgroups. The authors have done multiple subgroups analyses – stratifying by gender, education, marital status, etc. However, the authors have not tested for interaction. I would recommend testing for interactions when making comparisons regarding effects in different groups. If refraining from doing so the authors should be careful in their interpretations as they have not tested if the effect is indeed significantly different in their subgroups. For example: when the authors state “whereas the impacts were stronger for unmarried female workers (8.1 percentage points in job satisfaction and 6.9 percentage points in workhour satisfaction)” they have not actually tested if the impact is significantly stronger for unmarried female workers. It is fully possible that the effect size is significant in one group, and not in another without the two groups having significantly different effect sizes. It is also fully possible that two groups both have significant effects sizes, but that the effect sizes are still significantly different.*
- *Notably, if the authors use fixed affects analyses (only analyzing within effects) interaction between unchanging characteristics is not straight forward. But alternative methods can also take advantage of the longitudinal dataset and allow for interaction effects.*

**Response**: As the “Policy” indicator in the equation (1) represents difference-in-differences (DiD) estimates, interacting this Policy indicator with gender or socioeconomic status can be considered triple differences. Previous studies relying on triple differences frameworks acknowledged that this empirical method is for reducing biases by adding another control group unaffected by the policy change ([Palsson, 2017](https://www.sciencedirect.com/science/article/pii/S0047272717301810?casa_token=tjbMiI-K7pQAAAAA:zIvhVbZrAsNxGggJJFR0jNOjCmOvaSgOSqxxC8KtrPtIkbMjJ0di7oezP3Vq9WqBQLSQjg1B); [Olden and Moen, 2022](https://academic.oup.com/ectj/article/25/3/531/6545797)). Specifically, Palsson (2017) explored treatment heterogeneity by running separate regressions by each age group instead of interacting the DiD estimator with age groups. Following literature, we present estimates from separate DiD regressions to show heterogenous impacts of the policy.

Also, we agree that the magnitudes of the coefficients are not directly comparable across separate subgroups analyses. Our intention was to test differential impacts based on literature and our theoretical framework. To clarify, we rephrased the sentences in the **Results** section as follows:

“With respect to job and workhour satisfaction, male and female workers display different tendencies by marital status in Columns (3)-(4). Specifically, married male workers appear to have substantial gains in job (3.4 percentage points) and workhour satisfaction (3.9 percentage points) than unmarried peers, whereas among the female workers, the impacts were significant for unmarried female workers (8.1 percentage points in job satisfaction and 6.9 percentage points in workhour satisfaction) than married female workers."

- *Similarly, the authors state in the discussion “These findings suggest that socioeconomic status is among the main factors moderating the impact of working hours on workers’ well-being.” But they have not run a moderation analyses.*

**Response**: As we did not run a moderation analysis, we rephrased that sentence as follows to clarify;

“These findings suggest that the impact of working hours on workers’ well-being meaningfully varies by socioeconomic status.”

- *The authors should also acknowledge the consequences of testing each relationship in 26 (4 x 6 – 2) different subgroups. The share number of analyzes increases the possibility that the size and significance of the coefficients will vary by chance.*

**Response**: We added the following sentences to the **Discussion** section;

“Fourth, we acknowledge that our analysis includes multiple subgroups, and there is an increased possibility of finding significant estimates by chance due to small sample sizes within the subgroups. While this possibility would warrant further research with more complete data, the patterns of significant results support the findings from prior research.”

3. Language

- *Some minor languish errors e.g. “…confirm that workers’ weekly workhours generally reduced across” - sentence might be missing an “are”*

**Response**: We added “are” in that sentence. In addition, we have had several rounds of proofreading to correct typos.

**Reviewer #2**

- *The authors only consider ‘job satisfaction’, ‘work hours satisfaction’, and ‘leisure satisfaction’ as measures of quality of life. It is not clear why other aspects of life satisfaction, such as ‘satisfaction with family members or with social relationships’, and ‘satisfaction with living environment’, as well as ‘overall life satisfaction’ are not included in the analysis. Additionally, the differential impacts across different aspects of life satisfaction need to be interpreted more thoroughly.*

**Response**: We agree that the workers’ life satisfaction may not be fully measured by three indicators in our analysis. Following the suggestion, we ran additional regressions with other satisfaction measures available in KLIPS (family relationship, housing, and social life satisfaction), which appears in Appendix XX. The following point is added in the **Discussion** section;

“Third, this study is based on three satisfaction measures, including job, workhour, and leisure satisfaction. As these three measures may not fully represent the satisfaction of other aspects in one's life, we ran the analysis using three additional satisfaction measures in S4 Table (family relationship satisfaction, housing satisfaction, and social life satisfaction). We find that the satisfaction measure on family relationship increased significantly among women with low educational attainment, which aligns with our main findings. We also find that housing satisfaction significantly increased among female workers without precarious employment or without flexible employment.”

- *The endogeneity problem is a major concern in this study. The policy was implemented gradually from 2004 to 2011, and workers may have self-selected into establishments of certain sizes to benefit from the reduction of work hours. This could violate the exogeneity assumption of the model, as transitions between establishments before the policy was implemented may not be random. This issue needs to be addressed in the analysis.*

**Response**: To check the robustness of our study findings, we ran additional analyses with a restricted sample excluding those experienced changes in establishment sizes during the study period. The following was added at the end of the **Results** section;

“Furthermore, our results may be driven by workers who could self-select into establishments of particular sizes that benefit from the reduced work hours. In order to address the self-selection bias, we restricted our sample to workers who reported the same establishment size (within three categories: smaller than 50, 50-500, and 500 employees or more) during 2004-2011. This robustness check also found similar results (S2 Table) while standard errors are increased slightly due to the smaller sample size.”

- *I would like to see a more thorough discussion of the identification strategy. What variations are taken as exogenous using the staggered implementation of the new policy? When the biggest size group (1,000 or more workers) underwent the policy change, should we think of the rest of the size groups as the control, synonymous to a diff-in-diff strategy? I encourage the author to refer to the papers such as Goodman-Bacon (2021), and explain how we should interpret the results given that the firm-size groups received treatment at different times.*

**Response**: We appreciate the thoughtful comments. First, our control group consists of those never affected by the policy *and* those not affected by the policy yet. To clarify the control group in our analysis, we added the following sentence in the **Empirical** strategy section;

“Our control group consists of workers never affected by the policy and those not affected by the policy yet.”

With regard to Goodman-Bacon (2021), we acknowledge that the staggered implementation of the reduced workhour policy may warrant the use of a heterogeneity-robust difference-in-differences strategy (Goodman-Bacon, 2021; Sun and Abraham, 2020; Callaway and Sant’Anna, 2020; de Chaisemartin and D’Haultfoeuille, 2022). As de Chaisemartin and D’Haultfoeuille (2022) clearly pointed out, however, this generalized difference-in-differences typically requires a carefully selected control group. Given the nature of enforcing working hour reduction, it is natural to imagine that the workhour-reduction effects could have varied by workplaces (i.e. heterogeneity by share of highly-educated workers). As our data do not contain workplace identifiers, we are not able to detect changes in workhours of the control group that may have been affected by unobservable workplace-specific factors. For instance, probable existence of workers changing workplaces (without changes in the size of establishment) in the control group can challenge the validity of control group selection required to specify a generalized difference-in-differences estimation. Thus, we chose to present results from two-way fixed effects regressions as our main specification, and show additional regression results using an alternative difference-in-differences estimation suggested by de Chaisemartin and D’Haultfoeuille, (2022) in **Appendix** S5 Table. Generally, results are similar to the results from our main specification. We added the following in the **Discussion** section:

“Fifth, as our data do not contain workplace identifiers, we are not able to detect changes in workhours of the control group that may have been affected by unobservable workplace-specific factors. Given the nature of enforcing working hour reduction, it is natural to imagine that the workhour-reduction effects could have varied by workplaces (i.e. heterogeneity by share of highly-educated workers). Thus, we chose to present results from two-way fixed effects regressions as our main specification and show additional regression results using an alternative difference-in-differences estimation suggested by de Chaisemartin and D’Haultfoeuille (2022), which requires a carefully selected control group, in S5 Table. Overall, results are qualitatively similar to the results from our main specification.”
